# The Clustering of Adverse Childhood Experiences in the Avon Longitudinal Study of Parents and Children: Are Gender and Poverty Important?

**DOI:** 10.1177/0886260520935096

**Published:** 2020-07-08

**Authors:** Rebecca E. Lacey, Laura D. Howe, Michelle Kelly-Irving, Mel Bartley, Yvonne Kelly

**Affiliations:** 1 University College London, UK; 2 University of Bristol, UK; 3 Université Toulouse III—Paul Sabatier, France

**Keywords:** sexual assault, mental health and violence, domestic violence, child abuse

## Abstract

Previous research has demonstrated a graded relationship between the number of Adverse Childhood Experiences reported (an ACE score) and child outcomes. However, ACE scores lack specificity and ignore the patterning of adversities, which are informative for interventions. The aim of the present study was to explore the clustering of ACEs and whether this clustering differs by gender or is predicted by poverty. Data on 8,572 participants of the Avon Longitudinal Study of Parents and Children (ALSPAC) were used. ALSPAC is a regionally representative prenatal cohort of children born between 1991 and 1992 in the Avon region of South-West England. ACEs included parental divorce, death of a close family member, interparental violence, parental mental health problems, parental alcohol misuse, parental drug use, parental convictions, and sexual, emotional, and physical abuse, between birth and 19 years. Latent class analysis was used to derive ACE clusters and associations between poverty, gender, and the derived classes tested using multinomial logistic regression. Five latent classes were identified: “Low ACEs” (55%), “Parental separation and mother’s mental health problems” (18%), “Parental mental health problems, convictions and separation” (15%), “Abuse and mental health problems” (6%), and “Poly adversity” (6%). Death of a close family member and sexual abuse did not cluster with other adversities. The clustering did not differ by gender. Poverty was strongly related to both individual ACEs and clusters. These findings demonstrate that ACEs cluster in specific patterns and that poverty is strongly related to this. Therefore, reducing child poverty might be one strategy for reducing ACEs.

## The Importance of Adverse Childhood Experiences (ACEs)

Over the past two decades, the marked increase of research on ACEs has helped to highlight the importance of the early life social environment for life course health. Frequently investigated childhood adversities, such as child maltreatment and living with a parent with mental health problems, are all too common in the United Kingdom, like many other countries. For instance, a recent prevalence study in England showed that around half of adults reported at least one childhood adversity, most commonly living with a parent with a mental illness, parental separation/divorce, or having been verbally abused (Bellis et al., 2014). The high prevalence of ACEs is also reflected beyond survey data. For instance, in 2016/2017, there were 46.5 sexual offenses per 10,000 children aged 0 to 18 in the United Kingdom and the rate of recorded offenses has increased markedly from 18.7 sexual offenses per 10,000 children in 2012 (Bentley et al., 2018). Official figures are recorded offenses and as such are likely to be gross underestimates of the true population prevalence. Similarly, mental health problems are common, and it has been estimated that 68% of women and 57% of men with some degree of mental illness are parents of minors (Royal College of Psychiatrists, 2017).

## The Use of “ACE Scores” in Research

ACEs have been linked with an increased risk of numerous adverse outcomes across the life course. One of the most cited studies on the long-term effects of ACEs—the Kaiser Permanente Adverse Childhood Experiences study (Felitti et al., 1998)—showed that adults who experienced household dysfunction (living with a household member with substance misuse problems, mental illness, criminality, or witnessing interparental violence) or maltreatment (psychological, physical, or sexual abuse) in childhood had increased odds of many health outcomes, including ischemic heart disease, stroke, substance misuse, depression, and having attempted suicide. Moreover, the odds of these negative health outcomes increased in a dose-dependent manner with the number of adversities reported. This method of summing the number of adversities is known as the “ACE score” approach—an ACE score being the number of adversities reported. This approach has been replicated in hundreds of subsequent studies worldwide in relation to many different outcomes (e.g., Björkenstam et al., 2017; Dube et al., 2002; Hughes et al., 2017; Kelly-Irving, Lepage, Dedieu, Bartley, et al., 2013; Kelly-Irving, Lepage, Dedieu, Lacey, et al., 2013; Schilling et al., 2007). However, the limitations of ACE scores in both research and practice are becoming increasingly recognized (Lacey & Minnis, 2020).

## Exploring ACE Clustering

Adversities are known to co-occur, in that children who have one adversity are much more likely to report another. For instance, in the Kaiser Permanente study, between 81% and 98% of respondents who reported one ACE reported at least one other (Dong et al., 2004). Also, an analysis of serious case reviews of children who had experienced some kind of maltreatment found that more than half of those children had parents with mental health problems (Sidebotham et al., 2016). Consequently, it should not be assumed that adversities are isolated experiences but that each adversity experienced increases the chances of experiencing others. The ACE score approach is one simple method for dealing with the tendency of adversities to accumulate in a simple manner but has important limitations (Lacey & Minnis, 2020). These limitations include the underlying assumption that each adversity is equally important for outcomes, a disregard for the specific patterning of adversities and the importance of this for outcomes, and limited usefulness when investigating mechanisms linking adversities to outcomes (Lacey & Minnis, 2020). Consequently, the use of an ACE score alone is not useful for guiding interventions or policies; we also need to know more about the effects of separate adversities and about how, and which, adversities co-occur (Lanier et al., 2018). There is therefore a need to explore alternative methods for considering childhood adversities in research to identify how adversities cluster. As such, a recent report by the United Kingdom’s Early Intervention Foundation (Asmussen et al., 2020) has called for further research into how ACEs cluster. This is particularly key to investigate in longitudinal population samples as much of the prior research into ACEs has been conducted on unrepresentative samples, mainly of adults reporting ACEs retrospectively. Retrospectively reported ACE information is known to have important limitations (Baldwin et al., 2019).

There has been a recent growth of the application of alternative methods such as person-centered approaches (e.g., latent class analysis [LCA]) to explore the clustering of ACEs. LCA is a data reduction technique which allows researchers to identify clusters of individuals co-reporting similar ACEs. S. M. Brown and colleagues (2019) applied LCA to the National Survey of Child and Adolescent Well-Being II in the United States to a subgroup of participants who had experienced maltreatment, finding distinct clusters of other additional adversities in infancy, preschool age, school age, and adolescence. Furthermore, LCA has been applied to ACE data for university students in East Asia (Ho et al., 2020), finding three adversity clusters—“Low ACEs” (76.0%), “Household Violence” (20.6%), and “Household Dysfunction” (3.4%)—with those in the “Household Violence” group having particularly high risk of reporting depression and anxiety. In the British context, Denholm and colleagues (2013) applied LCA to child maltreatment information in the National Child Development Study (1958 British birth cohort), finding three classes (“Low risk of maltreatment” [66.9%], “Neglect only” [24.9%], and “High risk of abuse and neglect” [8.2%]). While the application of LCA to ACEs research has grown recently, this method has mainly been applied to maltreatment adversities rather than to a broader range of ACEs, such as parental separation and mental health problems. In the present study, we explore the clustering of broadly defined ACEs using LCA using a large population cohort from the United Kingdom.

## Poverty and ACEs

There has been little focus thus far in the adversity literature, and policy efforts on ACEs, on child poverty and inequality (Asmussen et al., 2020). Some studies have included the experience of poverty as an adversity, including as part of an ACE score (Appleton et al., 2017). However, we argue that poverty is different to many other psychosocial adversities, such as maltreatment and mental health problems, and instead we consider poverty to be an important risk factor for many childhood adversities. Indeed, recent research has shown that ACEs are strongly socioeconomically patterned at both the family (Walsh et al., 2019) and area level (Lewer et al., 2019). Consequently, there have been calls to focus on poverty and socioeconomic inequality as potential “causes” of ACEs (Institute of Health Equity, 2020; Metzler et al., 2017; Walsh et al., 2019).

In this study, we apply Townsend’s (1979) widely applied definition of relative poverty to indicate a level of resources, below the average family, which excludes full participation in society through living conditions, customs, and activities. Relative poverty is likely to put pressure on families who are unable to afford and provide the child with activities and living in conditions, which enable the child’s full participation in society. Indeed, the Family Stress Model illustrates how poverty has the potential to place strain on family relationships, potentially resulting in poor parental mental health and interparental conflict (Conger et al., 1992). Recent work showed that economic hardship was strongly related to ACE scores and this was mediated via poor maternal well-being (Liming, 2018). However, no studies have considered the role of poverty in predicting the *clustering* of childhood adversities—a focus of the present study.

## Gender and ACEs

Gender has also been relatively neglected as a potential determinant of ACE clustering. Girls are more likely to report maltreatment, particularly sexual abuse (Cutler & Nolen-Hoeksema, 1991; Dube et al., 2005; Hanson et al., 2008), and this is thought to have a larger effect upon the mental health of women (Gershon et al., 2008). A recent meta-analysis of gender differences in associations between child maltreatment and adult mental health only included five studies as few previous studies have stratified their analyses by gender (Gallo et al., 2018). The meta-analysis showed no gender differences of maltreatment on adult mental health, although the authors concluded that there is currently insufficient evidence. Further studies are therefore needed which consider whether the clustering of ACEs, including but not limited to maltreatment, differs by gender. In the present study, we explore whether there are gender differences in the clustering of ACEs in a large population cohort from the United Kingdom.

## Aim and Research Questions (RQs)

The aim of the present study was to explore the clustering of commonly investigated childhood adversities through the lens of gender and poverty. More specifically, we addressed three RQs:

**Research Question 1 (RQ1):** How do early life adversities cluster?
**Research Question 2 (RQ2):** Does the clustering of adversities differ for males and females?
**Research Question 3 (RQ3):** Does the clustering of adversities depend on poverty status (as defined by homelessness and problems affording heating, food, or accommodation)?

These three questions were addressed using a large longitudinal study with rich information on ACEs.

## Method

### Data

This study used data from the Avon Longitudinal Study of Parents and Children (ALSPAC), a prospective prenatal cohort from the Avon region of South-West England. This study recruited 14,541 women during pregnancy with expected delivery dates of April 1, 1991, to December 31, 1992 (71.8% of eligible pregnancies; Boyd et al., 2012; Fraser et al., 2013). The sample was boosted when the cohort children were approximately 7 years old with children with eligible birth dates who were not previously included in the study, resulting in a total of 15,247 eligible pregnancies and 15,458 fetuses. This total sample resulted in 14,775 live births, of whom 14,701 children were alive at 1 year of age. This study used information from surveys occurring between pregnancy and 19 years of age, including information reported by the cohort children’s main caregiver (usually the mother), mother’s partner, and the child themselves. ALSPAC has very rich, largely prospective data, on ACEs plus indicators of poverty during pregnancy (further details below), making it particularly suitable for the present study. Please note that the study website contains details of all the data that are available through a fully searchable data dictionary and variable search tool (http://www.bristol.ac.uk/alspac/researchers/our-data/).

### Measures

#### ACEs

Adversities included in this study were intrafamilial adversities commonly included in ACE score research. These were parental separation/divorce, death of a close family member (parent or sibling), parental convictions, parental drug use, parental alcohol misuse, parental mental health problems, interparental violence, physical abuse (parent–child), emotional abuse (parent–child), or sexual abuse (older child/adult child). Parental drug use was indicated by daily cannabis use or any use of hard drugs, such as amphetamines, heroin, cocaine, methadone, ecstasy, or barbiturates. Parental alcohol misuse was either daily binge drinking (>4 units per day) or self-reported alcoholism. Parental mental health problems were indicated by a score of 13 or more on the Edinburgh Postnatal Depression Scale indicative of “probable depression” (Boyd Le & Somberg, 2005), attempted suicide, and self- or partner-reported doctor consultations for schizophrenia, depression, or anxiety. These adversities were all prospectively measured from questionnaires with the cohort child’s mother or the mother’s partner, with the exception of sexual abuse; retrospective information on sexual abuse was reported in questionnaires completed by the young people themselves at ages 22 and 23 years (Harris et al., 2009), and was combined with prospectively collected data on sexual abuse, reported by the child’s mother across childhood. Cohort members were recorded as having experienced sexual abuse if this was recorded by either the mother or the cohort member themselves. Further information on the measures of early life adversities used and the questionnaires used to collect these data are shown in Online Appendix A.

#### Covariates

Covariates included in this study were gender and poverty. Consistent with Townsend’s definition of poverty, we decided to focus on material conditions rather than income measures as family income was not available until 21 months of age, precluding analyses between prior poverty and ACEs. Poverty was indicated by whether the cohort child’s parents reported difficulties in affording food, heating, or accommodation, or had recently been homeless at any point while pregnant with the cohort child. Any parent reporting any of these four difficulties was ascertained to be “in poverty” during pregnancy with the cohort child.

### Statistical Analyses

#### Missing data

Bias arising from missing data is a particular problem in longitudinal studies, as those who continue to participate over time tend to be more socially advantaged and healthier than those who drop out (Sterne et al., 2009). To reduce this bias, multiple imputation by chained equations was conducted to estimate missing values based on observed information, assuming that data were missing at random. Information on all adversities and covariates was imputed for those who had at least 50% of the potential 113 adversity variables observed across all waves of the study (*N* = 8,572), following a procedure previously used by Houtepen et al. (2018). Additional variables included in the imputation models were adversities from preceding and subsequent waves (e.g., during pregnancy and after age 19), as well as variables predictive of having missing information (e.g., social class, housing tenure, ethnicity, gestational age, and birthweight). Twenty imputed datasets were created, and all subsequent analyses were conducted on all imputed datasets, combining results using Rubin’s (1987) rules.

#### Clustering of adversities

We initially assessed the co-occurrence of ACEs by reporting the descriptive row percentages between ACEs and also constructing a tetrachoric correlation matrix to assess correlations between all binary ACE variables. We then applied a person-centered approach—LCA—with the aim of identifying subgroups of participants with co-occurring adversities. This method allows for the classification of individuals into groups or “classes” based on reported adversities which would typically be obscured by applying an ACE score approach. LCA models of two to five classes were estimated using the robust maximum likelihood (MLR) estimator. The number of classes in the final model was determined by comparing model fit statistics (Akaike’s Information Criterion [AIC], Bayesian Information Criterion [BIC], Sample Size Adjusted Bayesian Information Criterion [SSABIC], and likelihood ratio test). Lower AIC, BIC, and SSABIC values indicate a better fitting class solution, and entropy values closer to 1 show clearer distinction of classes (Celeux & Soromenho, 1996). Following many previous studies (e.g., S. M. Brown et al., 2019) and recommendations by Nylund et al. (2007), we give preference to BIC for determining the best class solution.

We followed the recommended three-step bias corrected method of LCA as this is the most optimal way of producing unbiased parameter estimates (Heron et al., 2015). In the first step, the classes were estimated in the absence of covariates (gender and poverty). Second, a categorical adversity cluster variable was created in which individuals in the dataset were assigned to their most likely ACE class based on their probability of class membership. Third, this adversity cluster variable became the dependent variable in a multinomial logistic regression, and associations between gender and poverty with the adversity cluster variable were estimated. Data management and multiple imputation were conducted in Stata Version 15.1 (StataCorp, 2017), and the LCA and subsequent regressions were conducted in Mplus Version 7.3 (L. K. Muthén & Muthén, 2012).

## Results

### Sample Characteristics

Table 1 shows the prevalence of each adversity in the observed and imputed data. The most commonly experienced adversity was mother’s mental health problems (52.1%), and just over a third of the sample reported parental separation or divorce (34.7%) by age 19. The least commonly reported adversities were the death of a parent or sibling (10.5%), physical abuse (11.2%), sexual abuse (9.4%), and parental drug use (9.5%). In addition, 15% of the cohort children were born to parents who had reported poverty during pregnancy and 51.1% of the sample was male.

**Table 1.** Description of the Study Sample (*N* = 8,572) and Comparison of Observed and Imputed Data.

Variables	Observed % (*n*)	Imputed %
Childhood adversities (0–19 years)		
Parental separation/divorce		
No	75.8 (1,481)	65.3
Yes	24.3 (474)	34.7
Death of close family member		
No	97.1 (877)	89.6
Yes	2.9 (26)	10.5
Interparental violence		
No	90.8 (1,182)	81.0
Yes	9.2 (120)	19.1
Physical abuse		
No	89.6 (3,025)	88.8
Yes	10.4 (352)	11.2
Sexual abuse		
No	91.1 (1,805)	90.6
Yes	8.9 (177)	9.4
Emotional abuse		
No	79.2 (2,690)	77.2
Yes	20.8 (705)	22.8
Parental convictions		
No	92.6 (1,091)	85.0
Yes	7.4 (87)	15.1
Mother’s mental health problems		
No	51.3 (1,379)	47.9
Yes	48.7 (1,307)	52.1
Father’s mental health problems		
No	76.1 (574)	67.1
Yes	23.9 (180)	32.9
Parental drug use		
No	93.4 (2,809)	90.5
Yes	6.7 (200)	9.5
Parental alcohol problems		
No	85.6 (853)	79.2
Yes	14.4 (144)	20.8
Gender		
Male	51.1	51.1
Female	48.9	48.9
Poverty		
No	85.3	85.0
Yes	14.7	15.0

*Note.* Only percentages are presented for multiply imputed data as the *n*s vary across the 20 imputed datasets.

### The Clustering of ACEs in ALSPAC

**Figure 1.**
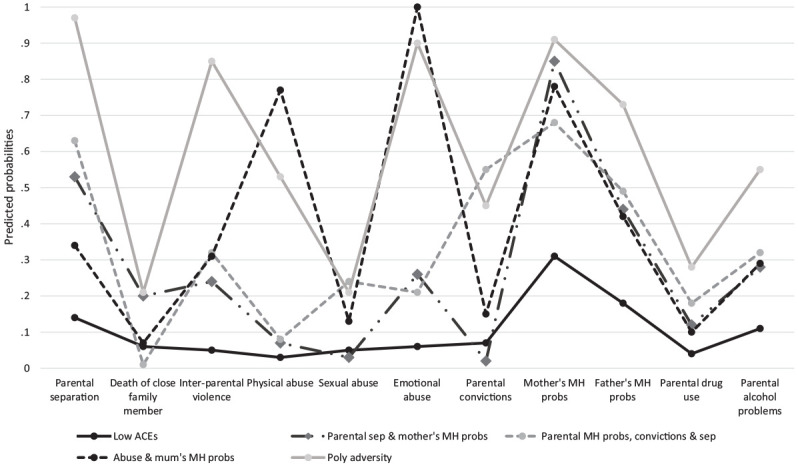
Probability of each adversity across the five latent classes. *Note.* MH = mental health; ACEs = adverse childhood experiences.

The descriptive co-occurrence of ACEs (row percentages) is shown in Table 2. In general, a high level of co-occurrence was observed in the ALSPAC sample. For instance, of the participants reporting parental separation or divorce, almost two thirds also reported maternal mental health problems, 45.5% reported paternal mental health problems, and around a third reported interparental violence, emotional abuse, or parental alcohol problems. This clustering is further reflected in statistically significant *p* values from a tetrachoric correlation matrix. The clustering of individuals based on their reporting of ACEs was tested using LCA and the most optimal class solution presented in Table 3 and Figure 1. A five-class solution was found to be the best fitting (see Online Appendix B). The largest class identified (“Low ACEs,” 54.4%) were people with a low probability of reporting any childhood adversity. The second largest class included participants reporting parental separation and mother’s mental health problems (“Parental separation and mother’s mental health problems,” 18.2%). The third class included participants who reported maternal and paternal mental health problems, parental convictions, and parental separation (“Parental mental health problems, convictions and separation,” 15.3%). The two smallest classes were comprised of participants who had experienced physical and emotional abuse and mother’s mental health problems (“Abuse and mother’s mental health problems,” 5.7%) and those reporting multiple adversities, including parental separation, interparental violence, physical and emotional abuse, parental mental health problems, and parental alcohol problems (“Poly adversity,” 6.4%). Death of a close family member, sexual abuse, and parental drug use did not appear to cluster with other adversities in this sample. Sensitivity analyses removing these adversities did not alter the class solution derived.

**Table 2.** Descriptive Co-Occurrence of Adversities (Row Percentages).

	Parental Separation	Death of Family Member	Interparental Violence	Physical Abuse	Sexual Abuse	Emotional Abuse	Parental Convictions	Mother’s Mental Health	Father’s Mental Health	Parental Drug Use	Parental Alcohol Problems
Parental separation	—	13.7	34.3	16.6	12.2	35.3	23.4	65.5	45.5	14.2	29.1
Death of family member	45.4***	—	33.7	12.5	10.3	31.4	17.0	62.5	37.6	12.7	26.0
Interparental violence	62.4***	18.5***	—	25.0	11.1	47.6	29.3	70.7	48.5	16.4	32.0
Physical abuse	51.3***	11.7*	42.4***	—	15.0	64.1	26.3	73.5	48.9	15.4	33.8
Sexual abuse	45.2***	11.5	22.6***	18.0***	—	32.4	21.8	68.4	39.9	15.2	32.0
Emotional abuse	53.6***	14.4***	39.6***	31.5***	13.3***	—	22.4	72.1	47.0	14.8	33.3
Parental convictions	54.8***	11.8	37.1***	19.6***	13.6***	34.0***	—	60.9	44.7	16.5	30.2
Mother’s mental health	43.5***	12.5***	25.8***	15.8***	12.3***	31.6***	17.6***	—	40.4	11.4	25.5
Father’s mental health	47.8***	11.9**	28.1***	16.7***	11.4***	32.6***	20.4***	64.0***	—	13.8	28.2
Parental drug use	51.9***	13.4	32.8***	18.3***	15.0***	35.8***	26.1***	62.6***	48.0***	—	36.5
Parental alcohol problems	48.5***	13.1***	29.3***	18.2***	14.4***	36.5***	21.9***	64.1***	44.6***	16.7***	—

*Note.* The *p* values represent level of significance of tetrachoric correlations and are shown for half of the table above and also apply to the corresponding correlations in the upper part of the above table.

* *p* < .05. **p < .01. ***p ≤ .001.

**Table 3.** Results of Latent Class Analysis of Early Life Adversities in ALSPAC.

	“Low ACEs”	“Parental Separation and Mother’s Mental Health Problems”	“Parental Mental Health Problems, Convictions and Separation”	“Abuse and Mental Health Problems”	“Poly Adversity”
% of sample in each class	54.4%	18.2%	15.3%	5.7%	6.4%
ACEs	Predicted Probabilities	Predicted Probabilities	Predicted Probabilities	Predicted Probabilities	Predicted Probabilities
Parental separation	.14	.53	.63	.34	.97
Death of close family member	.06	.20	.01	.07	.21
Interparental violence	.05	.24	.32	.31	.85
Physical abuse	.03	.07	.08	.77	.53
Sexual abuse	.05	.03	.24	.13	.21
Emotional abuse	.06	.26	.21	1.00	.90
Parental convictions	.07	.02	.55	.15	.45
Mother’s mental health problems	.31	.85	.68	.78	.91
Father’s mental health problems	.18	.44	.49	.42	.73
Parental drug use	.04	.12	.18	.10	.28
Parental alcohol problems	.11	.28	.32	.29	.55

*Note.* ALSPAC = Avon Longitudinal Study of Parents and Children; ACEs = adverse childhood experiences.

### Gender and ACEs

While the clustering of ACEs did not differ by gender (Table 4), we found differences in the reporting of individual ACEs for males and females (see Online Appendix C); females were more likely to report sexual abuse (odds ratio [OR] = 3.88, 95% confidence interval [CI] = [2.41, 6.22]) than males. However, females were less likely than males to report physical abuse (OR = 0.80, 95% CI = [0.66, 0.96]).

**Table 4.** Association Between Gender and Poverty With Adversity Clusters in ALSPAC.

	“Low ACEs”	“Parental Separation and Mother’s Mental Health Problems”	“Parental Mental Health Problems, Convictions and Separation”	“Abuse and Mother’s Mental Health Problems”	“Poly Adversity”
% of sample in each class	54.4%	18.2%	15.3%	5.7%	6.4%
	Odds Ratios (95% CI)	Odds Ratios (95% CI)	Odds Ratios (95% CI)	Odds Ratios (95% CI)	Odds Ratios (95% CI)
Gender (girls vs. boys)	Reference	1.26 [0.60, 2.65]	1.44 [0.59, 3.52]	0.71 [0.45, 1.11]	1.21 [0.88, 1.67]
Poverty (yes vs. no)	Reference	3.62 [1.26, 10.41]	4.51 [2.02, 10.06]	2.29 [0.96, 5.45]	9.15 [5.77, 14.51]

*Note.* ALSPAC = Avon Longitudinal Study of Parents and Children; ACEs = adverse childhood experiences; CI = confidence interval.

### Poverty and ACEs

Unlike gender, poverty was strongly associated with an increased odds of reporting every individual adversity type, with the exception of death of a close family member (see Online Appendix C). Associations were particularly strong between poverty and sexual abuse (OR = 2.38, 95% CI = [1.62, 3.52]), mother’s mental health problems (OR = 2.30, 95% CI = [1.93, 2.74]), and parental separation (OR = 2.63, 95% CI = [2.20, 3.14]). Poverty was also strongly associated with adversity clusters (Table 4). These associations were particularly pronounced for the “Poly adversity” class. For instance, children whose parents reported poverty in pregnancy were more than 9 times more likely to be in the “Poly adversity” cluster (OR = 9.15, 95% CI = [5.77, 14.51]) compared with the “Low ACEs” cluster than parents who did not report poverty. Poverty in pregnancy was also strongly associated with being in the ‘Parental separation and mother’s mental health problems’ (OR = 3.62, 95% CI = [1.26, 10.41]) and “Parental mental health problems, convictions and separation” (OR = 4.51, 95% CI = [2.02, 10.06]) classes compared with the “Low ACEs” class.

## Discussion

### Summary and Interpretation of Findings

#### RQ1: How do early life adversities cluster?

Using a large population dataset (the ALSPAC), we showed that adversities cluster and derived five classes of individuals reporting similar ACEs. More than half of the sample was included in the class reporting “Low ACEs,” almost a fifth of the sample in the class characterized by “Parental separation and mother’s mental health problems,” and a sixth of the sample reported the combination of “Parental mental health problems, convictions and separation.” Two smaller classes of approximately 6% reported “Abuse and mother’s mental health problems” and “Poly adversity.” The clusters derived in our study are consistent with those found in other work (Debowska et al., 2017; Ford et al., 2014; Lotzin et al., 2018; Merians et al., 2019; Mersky et al., 2017; Ross et al., 2018), although the key advances of the present study are that we find these classes in a longitudinal sample with mainly prospective data on ACEs.

One of the most striking findings with respect to ACE clustering in this study is the co-occurrence of parental mental health problems with other ACEs—most commonly parental separation and parental convictions but also emotional and physical abuse. In fact, parental mental health problems were part of the four ACE clusters we derived in this cohort. This may reflect the high prevalence of parental mental health problems compared with other ACEs in this sample. Yet the prevalence of parental mental health problems in ALSPAC is similar to other population cohorts, such as the UK-representative Millennium Cohort Study (Mensah & Kiernan, 2010). Parental mental health problems can place considerable strain on family relationships, potentially resulting in parental conflict, interparental violence, and parental divorce (Berg-Nielsen et al., 2002). These are important pathways through which parental mental health influences negative child outcomes (Duncan & Reder, 2000; Smith, 2004). The findings of our study in this respect might suggest that supporting parental mental health can reduce the effect of other ACEs. However, further research is needed to explore the directionality between different ACEs to explore whether parental mental health *precedes* or whether it in fact is a *consequence* of other ACEs.

In contrast, we found that the death of a close family member did not tend to cluster with other adversities. This is consistent with the findings of other studies; for instance, Mersky et al. (2017) found that death of a parent or sibling did not cluster with other commonly investigated ACE score adversities. Similarly, Lanier and colleagues (2018) applied LCA to the 2011/2012 National Survey of Children’s Health (NSCH) and found that the death of a parent did not cluster with other adversities. If a parent dies, then people are less likely to report any other parent-related adversities. It is also possible that close family member deaths do not cluster with other ACEs due to small numbers of cohort members experiencing this adversity, which means this ACE is statistically less likely to cluster with other ACEs. While the statistical clustering was not present here, it is conceivable that death of a close family member is linked to parental mental health problems and potentially other ACEs, such as substance misuse (Melhem et al., 2008).

In this study, sexual abuse also did not cluster with other adversities studied. This may reflect a difference in reporting mode as sexual abuse was mainly retrospectively reported by the cohort members at ages 22 and 23, whereas all other adversities were mainly prospectively reported by the cohort child’s mother or mother’s partner. For sexual abuse to be reported prospectively, at least one parent had to be aware and willing to disclose this information. The disclosure of sexual abuse is highly sensitive, and there is evidence to suggest that many victims of sexual abuse do not disclose the experience until many years later (McGuire & London, 2020). Given the highly sensitive nature of sexual abuse, it is also less likely to be reported at all (Finkelhor, 2005).

#### RQ2: Does the clustering of adversities differ for males and females?

We found that females were more likely to report sexual abuse and males were more likely to report physical abuse, and this concurs with other studies which have collected this information (G. Brown & Anderson, 1991; Dube et al., 2005; Martin et al., 2004). We also found no gender differences in the way in which ACEs cluster in this study. Recent work by Liu et al. (2018) and Lanier et al. (2018) showed no gender differences in latent class membership using the 2011–2012 NSCH. We extend this previous work, showing similarities in findings in the United Kingdom in a prospective longitudinal study. In our exploration of ACEs beyond child maltreatment, we also showed that other ACEs, such as parental separation, substance misuse, and interparental violence, do not differ by gender. This makes sense given that these are parent-focused ACEs, rather than parent–child ACEs in which the characteristics of child are likely to play little influence. Hence, this is likely why we also show no differences in the clustering of ACEs by gender here.

#### RQ3: Does the clustering of adversities depend on poverty status?

Poverty was found to be strongly related to both individual adversities, particularly parental separation, sexual abuse, and maternal mental health problems. This echoes Conger’s Family Stress Model (Conger et al., 1992). This model shows how financial hardship can place immense strain on parental relationships, increasing the risk of interparental conflict, violence, and separation. Indeed, transition into poverty has been shown as an important risk factor for maternal mental health problems in the UK-representative Millennium Cohort Study (Wickham et al., 2017).

Cohort members whose parents had experienced poverty during pregnancy were also much more likely to be in one of the adversity clusters rather than in the “Low ACEs” group, with the exception of the “Abuse and mother’s mental health problems” class. The relationship between the experience of poverty and being in the “Poly adversity” cluster compared with the “Low ACEs” cluster was found to be particularly strong. Previous studies have also shown that people with fewer economic resources tend to report higher ACE scores (Liming, 2018; Metzler et al., 2017; Steele et al., 2016). Again in line with the Family Stress Model, previous work has shown that the relationship between economic hardship and a high ACE score was mediated by poorer maternal well-being (Liming, 2018). We ran a sensitivity analysis to compare the association between poverty and “Poly adversity” with that between poverty and an ACE score of 4+, taking the approach of hundreds of previous studies that have instead used the ACE score approach. We found that the relationship between poverty and “Poly adversity” was stronger than that observed between having an ACE score of 4+ (OR = 6.87, 95% CI = [5.35, 8.81]), therefore suggesting that the specific patterning of adversity is important beyond the number of ACEs reported (Lanier et al., 2018).

### Methodological Considerations

This study has a few limitations which should be borne in mind when interpreting the findings. First, the entropy level (0.67) of our five-class solution was lower than in some other papers, suggesting that our adversity classes were less distinct in this dataset. However, this entropy value is consistent with several other studies using the same analytical approach (S. M. Brown et al., 2019; Lanier et al., 2018; Liu et al., 2018; Merians et al., 2019; Rebbe et al., 2017). There has been recent discussion about what constitutes a “good” entropy value, and 0.8 is frequently cited although a marginally lower entropy value does not mean the model is not useful (B. O. Muthén, 2018). Furthermore, it is recommended that entropy should not be used as a model selection tool (Masyn, 2013). Correspondingly, we conservatively allocated individuals to a class based on their estimated probabilities of class membership rather than exclusively allocating each individual to a single class to reflect the increased uncertainty of true class membership. Second, one of the main criticisms of the LCA approach is that the results are dataset-specific. However, we found a high level of consistency between the clusters we derived and those derived in other studies utilizing different samples. Third, we chose to focus on poverty in pregnancy to ascertain temporal ordering. However, it is possible that the birth of the cohort member (and further children) stretched family finances further, but we do not model the interrelationships between family poverty and ACEs in the present study. Fourth, the ALSPAC sample was not randomly selected and therefore may not be representative of the national population.

This study also has a number of strengths. First, we used a large population sample from the United Kingdom to explore the clustering of ACEs. Second, ALSPAC has rich prospective and retrospective information on ACEs, unlike most longitudinal population studies, enabling appropriate examination of the RQs in this study. Third, missing data on adversities and covariates were accounted for using multiple imputation, thereby reducing the bias attributable to attrition and nonresponse. Finally, by applying an LCA approach, we were able to explore the person-centered clustering of adversities and to apply this to broader ACEs than just child maltreatment that have largely been focused on using this analytical approach. This method enabled us to explore the clustering of ACEs hypothesis free. This is appropriate in a field in which this method has been little applied thus far (Debowska et al., 2017).

## Conclusions and Implications

In summary, we showed that there is clustering of ACEs in the ALSPAC cohort, and that five clusters can be derived using an LCA approach. Parental mental health problems were highly prevalent in this cohort and consequently were a key component of each of the ACE clusters. Given this, parental mental health improvement should be a priority. The relationship between parental mental health problems and other ACEs is likely to be complex, and further research is needed as to whether parental mental health precedes or follows other ACEs.

The clustering of adversities did not differ by gender. Poverty was strongly associated with all adversity clusters and more strongly related to the “Poly adversity” cluster than to an ACE score, suggesting that the specific combination of ACEs in that cluster was more important than the number of ACEs. Consequently, poverty alleviation may be a critical element of ACE reduction, and there have been criticisms that many policies and programs aiming to reduce ACEs and their consequences ignore the role of poverty (Lacey & Minnis, 2020; Walsh et al., 2019). Income supplementation, conditional cash transfers, and housing interventions have been shown to be particularly effective for ACE reduction, particularly for reducing rates of child maltreatment and parental substance misuse (Courtin et al., 2019). While these upstream approaches have been effective, there have been recent calls for a whole-systems approach to ACE reduction and prevention, largely focusing on reducing the number of families living in poverty (Institute of Health Equity, 2020).

The implications of this study are that ACE scores may not be the most appropriate method to account for the clustering of adversities. The ACE score approach assumes that all adversities included are correlated (Evans et al., 2013), yet three (death of a close family member, parental substance misuse, and sexual abuse) were not in this study. This suggests that an ACE score approach would be inappropriate in this context. The ACE score approach also ignores the specific patterning of adversities, which we show to be important. Further research is needed to test associations of the derived adversity classes with child outcomes, comparing their predictive validity with that of ACE scores. Future work is also needed to assess the temporal sequencing of multiple adversities to identify which ACEs most commonly occur first. This would enable the targeting of interventions to the specific ACEs, which tend to precede or predict others.

## Supplemental Material

sj-pdf-1-jiv-10.1177_0886260520935096 – Supplemental material for The Clustering of Adverse Childhood Experiences in the Avon Longitudinal Study of Parents and Children: Are Gender and Poverty Important? Click here for additional data file. Supplemental material, sj-pdf-1-jiv-10.1177_0886260520935096 for The Clustering of Adverse Childhood Experiences in the Avon Longitudinal Study of Parents and Children: Are Gender and Poverty Important? by Rebecca E. Lacey, Laura D. Howe, Michelle Kelly-Irving, Mel Bartley and Yvonne Kelly in Journal of Interpersonal Violence
