# Supplementary material for: The Clustering of Adverse Childhood Experiences in the Avon Longitudinal Study of Parents and Children: Are Gender and Poverty Important?
Source: J Interpers Violence. 2020 Jul 8;37(5-6):2218–41. doi: 10.1177/0886260520935096 (PMC8918866; doi:10.1177/0886260520935096)
Supplement: sj-pdf-1-jiv-10.1177_0886260520935096 – Supplemental material for The Clustering of Adverse Childhood Experiences in the Avon Longitudinal Study of Parents and Children: Are Gender and Poverty Important? [file sj-pdf-1-jiv-10.1177_0886260520935096.pdf]

Appendix A Information on childhood adversities used and their collection in ALSPAC

Adversity	Information adversity derived from	Respondent	Ages used																					
			8w	8m	1y 6m	1y 9m	2y 5m	2y 9m	3y 6m	3y 11m	4y 9m	5y 1m	5y 9m	6y 1m	6y 9m	8y 6m	9y 2m	11y 2m	12y 1m	16y	17y	19y	22y	23y
Parental separation or divorce	Mother separated/divorced from partner since xxx	Mother																						
	Partner separated/divorced from partner since xxx	Mother's partner																						
	Parents divorced or separated since xxx	Study child																						
Death of close family member	Death of father	Mother																						
	Death of a child	Mother																						
	Death of mother	Mother's partner																						
	Death of a child	Mother’s partner																						
	Death of a parent	Study child																						
	Death of a sibling	Study child																						
Interparental violence	Mother's partner physically cruel to mother since xxx	Mother																						
	Mother hurt partner since xxx	Mother																						
	Mother physically cruel to partner since xxx	Mother's partner																						
	Mother's partner physically cruel to mother since xxx	Mother’s partner																						
Physical abuse	Mother been physically cruel to child	Mother																						

	Mother's partner been physically cruel to child	Mother																						
	Child physically hurt by someone	Mother																						
	Partner been physically cruel to child	Mother's partner																						
	Partner's partner been physically cruel to child	Mother's partner																						
Sexual abuse	Child sexually abused since xxx	Mother																						
	Touched in sexual way by older child or adult	Study child																						
	Forced into sexual activity by older child or adult	Study child																						
	When growing up someone molested child sexually	Study child																						
Emotional abuse	Mother been emotionally cruel to child	Mother																						
	Mother's partner been emotionally cruel to child	Mother																						
	Partner been emotionally cruel to child	Mother's partner																						
	Partner's partner been emotionally cruel to child	Mother's partner																						
Parental convictions	Mother convicted of a crime since xxx	Mother																						
	Mother's partner convicted of a crime since xxx	Mother's partner																						

Mother's mental health problems	Edinburgh Postnatal Depression score >12	Mother																						
	Suicide attempt since xxx	Mother																						
	Mother had schizophrenia since xxx	Mother																						
	Mother sought help for depression	Mother																						
	Mother sought help for depression	Mother's partner																						
	Mother sought help for schizophrenia	Mother’s partner																						
Father's mental health problems	Edinburgh Postnatal Depression score >12	Mother's partner																						
	Suicide attempt since xxx	Mother’s partner																						
	Sought Dr for depression	Mother’s partner																						
	Sought Dr for schizophrenia	Mother’s partner																						
	Mother's partner had schizophrenia since xxx	Mother																						
	Suicide attempt since xxx	Mother																						
	Mother's partner sought Dr for depression	Mother																						
Parental drug use	Uses cannabis daily	Mother																						

	Uses amphetamines, cocaine, heroin, methadone, ecstasy, other hard drugs since xxx	Mother																						
	Uses cannabis daily	Mother's partner																						
	Uses amphetamines, cocaine, heroin, methadone, ecstasy, other hard drugs since xxx	Mother's partner																						
Parental alcohol problems	Drinks more than 4 units daily	Mother																						
	Reported an alcohol problem	Mother																						
	Drinks more than 4 units daily	Mother's partner																						
	Reported an alcohol problem	Mother's partner																						

Appendix B – Comparing different latent class solutions

	**5 class solution**	**4 class solution**	**3 class solution**	**2 class solution**
% of participants in each class				
Class 1	**6.4**	29.2	26.3	26.5
Class 2	**15.3**	7.1	12.1	73.5
Class 3	**5.7**	6.9	61.7	
Class 4	**54.4**	56.8		
Class 5	**18.2**			
Model fit indices				
BIC	**84907.5**	84942.7	85046.9	85327.2
SSABIC	**84720.0**	84793.3	84935.7	85254.1
AIC	**84491.2**	84611.0	84800.0	85164.9
Log likelihood	**-42186.6**	-42258.5	-42365.0	-42559.5
Entropy	**0.65**	0.67	0.64	0.69

Appendix C Associations between gender and poverty with childhood adversities in ALSPAC

	**Gender<br>OR (95% CI)<br>Girls vs boys**	**Poverty<br>OR (95% CI)<br>Poverty vs no poverty**
Parental separation	1.06 (0.93, 1.20)	**2.63 (2.20, 3.14)**
Death of close family member	1.19 (0.79, 1.77)	1.13 (0.48, 2.66)
Inter-parental violence	0.98 (0.83, 1.15)	**2.02 (1.61, 2.54)**
Physical abuse	**0.80 (0.66, 0.96)**	**1.77 (1.40, 2.24)**
Sexual abuse	**3.88 (2.41, 6.22)**	**2.38 (1.62, 3.52)**
Emotional abuse	0.92 (0.83, 1.03)	**2.00 (1.70, 2.35)**
Parental convictions	1.16 (0.97, 1.39)	**1.73 (1.22, 2.47)**
Mother's mental health problems	1.01 (0.92, 1.12)	**2.30 (1.93, 2.74)**
Father's mental health problems	1.07 (0.92, 1.24)	**1.81 (1.29, 2.55)**
Parental drug use	1.02 (0.86, 1.22)	**2.26 (1.83, 2.80)**
Parental alcohol problems	1.15 (0.96, 1.38)	**1.64 (1.27, 2.11)**
